# Mood disorders and suicide: pilot study on postmortem toxicologic evidence and adherence to psychiatric therapy by determining blood levels of medications

**DOI:** 10.1007/s00414-024-03327-8

**Published:** 2024-09-14

**Authors:** Stefano Tambuzzi, Guido Travaini, Orsola Gambini, Federica Collini, Lorenzo Ginepro, Francesco Attanasio, Lorenzo Fregna, Federica Zucca, Domenico Di Candia, Alida Amadeo, Cristina Colombo, Alessio Battistini, Cristina Cattaneo

**Affiliations:** 1 Institute of Forensic Medicine, Department of Biomedical Sciences for Health, University of Milan, Luigi Mangiagalli, 37, Milan, 20133 Italy; 2 University Vita-Salute San Raffaele, Milan, 20132 Italy; 3 Department of Biomedical Sciences for Health, San Paolo Hospital, University of Milan, Milan, 20142 Italy; 4 Department of Health Sciences, University of Eastern Piedmont Amedeo Avogadro, Novara, 28100 Italy; 5 Department of Biosciences, University of Milan, Milan, 20133 Italy; 6 Department of Biomedical, Surgical and Dental Health Sciences, University of Milan, Milan, Italy

**Keywords:** Suicide, Mood disorders, Depression, Bipolar disorder, Therapeutic compliance, Psychiatric therapy, Toxicological analysis

## Abstract

Suicide is one of the leading causes of death today, and among all mental illness, mood disorders account for one of the main risk factors. It is well known and proven that suicides are very common among people undergoing treatment and prescribed psychiatric medication. So far, however, there have only been a few studies dealing with this particular phenomenon. For this reason, autopsy patients who died by suicide, suffered from a mood disorder, and were known to be taking psychiatric medication at the time of death were selected for this study. The blood and urine samples taken during the autopsy underwent toxicological analysis and the results were compared with the prescribed therapy. A total of 22 people were included in the study: 12 presenting with depression and 10 with bipolar disorder. The toxicological analysis revealed that only 6 cases (27%) showed a qualitative match with the prescribed medication. In 5 cases (22.7%) the medication was only partially complied with and in 11 cases (50%) it was not complied with at all. Furthermore, even when medication was present, the value was often below the therapeutic range. Overall, more than 70% of the test subjects adhered to their medication only partially or not at all. Since treatment adherence is considered as a key factor in reducing the risk of suicide, this inevitably raises relevant clinical and forensic questions. Against this background, prospective monitoring of post-mortem medication levels in suicidal individuals and synergistic collaboration between clinicians and forensic pathologists could help to evaluate the effectiveness of specific medical interventions, highlight existing critical problems and develop new approaches to suicide prevention.

**Supplementary Information:**

The online version contains supplementary material available at 10.1007/s00414-024-03327-8.

## Introduction

Suicide is one of the leading causes of death today and is a widespread public health and social impact problem, with around 800,000 deaths per year worldwide. The entity of this occurrence has also been recognized by the World Health Organization (WHO), which has identified suicide as one of the most important research topics for studying its causes and identifying effective prevention measures [1]. In this context, it is generally recognized that psychiatric disorders are the main risk factor for suicide, with the population with such disorders having a 10- to 30-fold increased risk compared to the general population [2]. The most common psychiatric diagnoses associated with suicide include mood disorders, substance abuse, schizophrenic spectrum disorders, personality disorders, and eating disorders. Among all of these, mood disorders have an increasing impact on the global burden of disease and are the third leading cause of disability worldwide [3]. These include Major Depressive Disorder (MDD) and Bipolar Disorder (BD), in which suicidal ideation is a key symptom according to the Diagnostic and Statiscal Manual of Mental Disorders (DSM-5) [4] and occurs in more than 50% of cases [5]. This explains very well why there are so many suicide attempts among these people in the course of their lives, even if the percentages in the various studies are very different. In any case, the literature indicates that between 25 and 60% of patients with bipolar disorder attempt suicide at least once in their lifetime, with between 3 and 20% being completed. In patients with MDD, the prevalence of suicide attempts appears to be somewhat lower and is estimated at between 2 and 15%. However, when considering the high prevalence of this disorder in the global population (4.4%) [6], hundreds of millions of people are affected [1, 6–8]. Extremely significant is the fact that 65 to 90% of all suicides are associated with a mood disorder (especially MDD), with a higher prevalence in cases where no specific drug therapy for the psychiatric disorder has been prescribed or properly taken [9]. In this context, many developed countries have introduced national suicide prevention plans, such as screening campaigns that focus on general practitioners detection of depression and better treatment strategies. Data from many countries shows a link between increased antidepressant prescribing and a decrease in suicide rates [10]. Many other classes of medications have been proposed as effective strategies for suicide prevention in psychiatric disorders in general, including lithium, mood stabilizers, antipsychotics such as clozapine, and benzodiazepines. The prevention of suicidal behavior and better management of impulsivity and disinhibition in general [10, 11] should be the aims of each mental health care system. Impulsivity and disinhibition are indeed characteristic of people with bipolar disorder and could be one of the main factors for the higher suicide rate in patients with this disorder compared to patients with depression [12]. Overall, the effect of antidepressants on suicide has been controversial in some older reports concerning about possible side effects, such as the “increase in suicides” and the general toxicity of some antidepressants [13–16]. In particular, antidepressant use has been associated with an increased risk of suicidality and suicidal behavior in children, adolescents, and young adults under 25 years of age [17, 18]. Some more recent literature evicedence suggests that also benzodiazepine use may increase the risk of suicide as they can influence the patient’s degree of impulsivity and disinhibition [19]. However, numerous studies have been conducted and, based on recent meta-analyses, it seems clear that the latest evidence in the literature converges to identify pharmacologic therapy as the mainstay for the management of suicide risk in patients with mood disorders [20, 21]. Although these patients are often treated with psychopharmacological therapies either in hospitals or out-patient services for mental health, it is a common experience that many suicides occur precisely among these people. This is a particularly critical point that requires further investigation. Indeed, the literature on postmortem toxicologic analysis in decedents with psychiatric disorders has so far focused mainly on the detection of drugs, as these are potentially lethal in case of accidental or intentional overdose. However, there are no studies aimed at analyzing postmortem biological samples from individuals with psychiatric disorders who died by suicide and were prescribed drug therapy to assess the victims’ compliance to treatment at the time of death. According to the WHO and scientific literature, medication compliance (synonym: Adherence) refers to the degree or extent of conformity to the recommendations about day-to-day treatment by the provider with respect to the timing, dosage, and frequency. So in other words, it may be defined as “the extent to which a patient acts in accordance with the prescribed interval, and dose of a dosing regimen” [22, 23]. In the clinical setting, the only way to assess a patient’s adherence to therapy is by dosing the drug in biological samples. For this purpose, blood is the most commonly used substrate as it reflects the concentration of the drug in the circulation at a given time and is a valid measure of the adequacy of the drug dose administered [24, 25]. There are numerous studies in the literature describing how the measurement of plasma levels of drugs such as carbamazepines [25], antiepileptics [26], antidepressants [27] and antipsychotics [28] has been used as an indirect indicator for assessing patient adherence to treatment. This applies in particular to psychiatry, as the adherence of patients with psychiatric disorders to treatment is a well-known problem. A recent meta-analysis examined this very problem by dividing various psychiatric disorders into subgroups to identify differences in patient adherence to treatment [29]. It was found that in patients with psychotic disorders, non-adherence to treatment was 49% based on 35 studies totaling 63,975 cases, with rates varying by geographic location: 48% in Africa, 48% in North America, 49% in Europe and Asia. In schizophrenic patients, non-compliance increased to 56% based on 9 studies with a total of 2643 cases. In the affective disorders, treatment adherence for major depression was 50% based on 16 studies with a total of 42,225 cases and 44% for bipolar disorder based on 10 studies with a total of 73,250 cases. It is thus clear that the problem of poor treatment adherence among people with psychiatric disorders affects millions of people worldwide, many of whom die by suicide. It is precisely in these cases that postmortem analysis of biological samples could be important from various points of view: clinical, forensic and epidemiological, as it can be used to derive data closely related to the geographical area under study. Currently, no such approach has never been reported in the literature and, therefore, an attempt was made to at least partially address this gap by conducting a pilot study with a very multidisciplinary approach. The aim was to carry out postmortem toxicological examinations of blood and urine samples from people with mood disorders who were undergoing pharmacological treatment and had died by suicide, and to compare these results with the psychiatric therapy prescribed to them. In other words, the aim was to investigate the extent to which these patients were adherent to treatment at the time of their suicide by assessing the presence or absence and concentration of medications in the blood. At the same time, a comprehensive chemical-toxicological post-mortem analysis of these patients was carried out. We would therefore like to point out that it was not the aim of this study to assess whether the prescribed psychiatric therapy was correct or not from a clinical point of view.

## Materials and methods

### Settings and cases extrapolation procedure

A retrospective cross-sectional study was conducted at the Institute of Forensic Medicine in Milan, one of the largest cities in Italy, where numerous autopsies are routinely performed at the request of judicial or health authorities. Before each autopsy, an interview is held with the victim’s relatives and all reported anamnestic information, such as previous and current illnesses, drug treatments and the use or abuse of psychoactive substances, is recorded and entered into an internal database. In the case of suicide victims, it is investigated whether and for how long the deceased was treated by mental health professionals, either in hospitals or outservices for mental health. It should be noted that the interview with family members is a critical opportunity to gather essential information about the deceased, for whom medical records are usually unavailable prior to the autopsy. Generally, medical records can only be formally requested after the autopsy.

For this study, the internal database was retrospectively analyzed to extrapolate all individuals (including both adults and minors) who died by suicide in the last 10 years (2012–2022) and simultaneously suffered from a diagnosed mood disorder: Major Depressive Disorder (MDD) or Bipolar Disorder (BD). The internal database allows for the identification and extrapolation of cases of interest using specific search terms. It should be noted that in all autopsy cases, the final determination of the manner of death - whether suicide, homicide, accident or natural causes - is based on the integration of pathological and forensic findings with circumstantial and investigative data collected and provided by law enforcement officers. In the absence of sufficient evidence, the manner of death may be classified as presumed or undetermined. Consequently, only those cases that were definitively classified as suicide were included. Regarding the psychiatric disorders (MDD or BD), at this preliminary stage of case extrapolation, we relied on information reported by family members and recorded in the database.

**Fig. 1:**
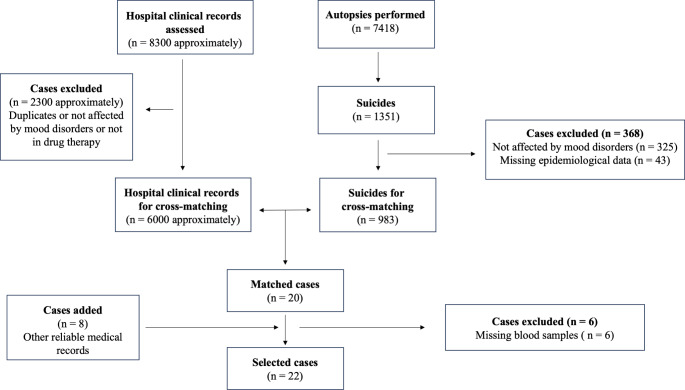
Flowchart for the extrapolation of cases of interest

The generalities of the extrapolated individuals (no. = 983 in total, Fig. 1) were then compared with the internal databases of two of the major hospitals in the city with which a collaboration was established for this study: San Raffaele Hospital and ASST Santi Paolo e Carlo Hospital, which are characterized by a large catchment area and tens of thousands of emergency admissions per year; in addition, both have a psychiatric department and, in the case of San Raffaele Hospital, a mood disorder unit. In this way, it was possible to confirm the psychiatric diagnosis of mood disorder and obtain reliable health records on the last psychiatric drug therapy prescribed to the patients (which medications and with what dosage). There was indeed a need for proven information, and relying solely on information from family members (both for disease diagnosis and prescribed therapy) would have biassed the study, as it may be incomplete. A total of about 8300 clinical records were examined (Fig. 1).

Only subjects for whom the last prescribed therapy could be traced with certainty on the basis of the medical records (no. = 20, Fig. 1) were selected for the study.

The remaining cases, which were extrapolated from the Institute of Forensic Medicine database and had no match in the hospital databases, were carefully evaluated, selecting only those cases for which equivalent and demonstrably reliable health data were available (no. = 8, Fig. 1). These were people who were certainly receiving psychiatric drug therapy, as they were being treated in other mental health centers or by private psychiatrists. When interviewing the family members of the deceased before the autopsy, it may occasionally happen that medical records are issued and thus acquired.

We would like to point out that the authors of this study have not made any psychiatric diagnoses, but have relied on the diagnoses made by psychiatrists and listed in the latest official medical documentation. In Italy, psychiatric diagnoses are made on the basis of the Diagnostic and Statistical Manual of Mental Disorders. Therefore, for the diagnoses of psychiatric disorders of the individuals selected for this study, it can be stated that the international classification referred to is the DSM-5 [4].

For all selected cases (no. = 28, Fig. 1), it was subsequently checked whether biological samples, in particular femoral blood, were stored on the premises of the Institute of Forensic Medicine for toxicological analysis. It is common practice that biological samples are taken at autopsy and properly frozen. Blood was selected as the most appropriate substrate for investigating recent medication intake and assessing the concentration in the blood at a specific time point (in this study at the time of suicide). In 6 cases the blood sample was missing, so they were excluded (Fig. 1).

Furthermore, in each of the selected cases, when available, urine was also subjected to toxicological analysis, as although it is not suitable for the assessment of circulating active medications, it nevertheless allows an examination of relatively recent ingestion (hours to days, sometimes weeks), highlighting those substances that are no longer detectable in the blood [30]. It was therefore assumed that urine could be particularly useful for the evaluation of drugs with a short half-life in the blood. In other words, urine alysis could have extended the assessable toxicologic window of the cases, as it was the only substrate for investigating semi-recent intakes, as hair was not retrospectively available in these cases.

Finally, the following epidemiologic information was collected for all selected cases included in the study: gender, age, nationality, comorbidities, previous suicidal ideation, previous suicide attempts, marital status and occupational status, and suicide modality. As far as comorbidities are concerned, those listed in the medical records were taken into account. For each of these cases, additional forensic information such as the state of body preservation and the post-mortem interval (time of death, last seen/heard alive) as well as the autopsy date were recorded in order to better interpret the toxicological findings.It should be noted that corpses are kept in refrigerated storage from the time of death (witnessed deaths) or discovery in order to preserve their state of preservation.

The entire flowchart of the case extrapolation procedure is shown in Fig. 1.

### Exclusion criteria

Exclusion criteria were: (i) missing or incomplete sociodemographic information such as sex, age, nationality, marital status (married/single/divorced/widowed) and occupational status (employed/unemployed/retired); (ii) manner of death other than suicide; (iii) missing medical diagnosis of the mood disorder the victim was suffering from; (iv) missing medical records of the last psychiatric drug treatment at the time of suicide; and (v) missing femoral blood sample. The absence of a urine sample was not considered an exclusion criterion, as the analysis of blood was the primary goal for the pursuit of the study objective. After all extrapolations were completed, selected cases were anonymized and assigned a unique numerical identifier before the subsequent toxicological analyses were performed.

### Toxicological analysis

The blood and urine samples of the selected cases were subjected to toxicological analysis to determine the concentration of volatile and non-volatile compounds and to quantify the substances detected. The volatile organic compounds were analyzed using the headspace technique, and the internal standard method was used for quantitative evaluation. For the evaluation of ethanol content, a calibration curve was established with standard solutions of ethanol in water at increasing concentrations. Non-volatile organic compounds were sought by extraction procedures with suitable organic solvents and analyzed using the HPLC-MS/MS system (High-Performance Liquid Chromatography - Tandem Mass Spectrometry). The obtained uptake spectra were processed and interpreted using the “Tracefinders 5.1” software, which allows comparison with substances from the inclusion list used at the toxicology laboratory of the Institute of Forensic Medicine in Milan and published in the online database “m/z cloud”. The lithium analysis was performed using the ICP-MS technique (inductively coupled plasma mass spectrometry). Toxicological analyses were conducted according to national [31] and international [32] guidelines, and the detail of the laboratory methodological approach is given in the supplementary materials (S1). Finally, the results were compared with the ongoing drug therapy at the time of death, as reported in the acquired medical records. The concentrations of the drugs detected in the blood were interpreted on the basis of literature references both for therapeutic ranges [33–38] and half-lives [39–44].

### Statistical analyses

Statistical analyses were performed with Stata 18 (StataCorp 2023. College Station, TX: StataCorp LLC). Groups were compared on sociodemographic, clinical, and suicide-related variables using standard tests. The Shapiro-Wilk test was carried out to determine a Gaussian distribution. As the ages of the subjects in both groups were normally distributed, a Student’s t-test was performed for independent variables to test whether there were statistically significant differences between the mean ages of the two groups. The null hypothesis in this case was that there were no statistically significant differences between the mean ages of the two groups, with the p-value set at ≤ 0.05. For the other socio-demographic (marital status and occupational status) and clinical/suicide-related (compliance and care facility) parameters, the chi-square test (χ^2^) and the Z-score test were used, with a statistically significant p-value set at ≤ 0.05 in both cases.

## Results

After the case extrapolation procedure as reported in Fig. 1 and 22 people (11 men and 11 women) aged between 28 and 83 years were included in the study. No minors were present in the selected cases. They died by suicide with the following modalities: fall from a great height (10 cases, 45.5%), hanging (7 cases, 32%), sharp force trauma (2 cases, 9%), gunshot wound (1 case, 4.5%), plastic bag suffocation (1 case, 4.5%) and acute drug intoxication (1 case, 4.5%). All bodies appeared to be in a good state of preservation, some had an initial greenish putrefactive discoloration of the abdomen, and the autopsy was always performed no more than a few days after body discovery (Table 1).

**Table 1:** Details of the main epidemiological, health and forensic information of the cases selected for the study (H: hospital; PP: private psychiatrist; OPC: out-patients mental health center)

	Gender	Age	Mood disorder	Care facility	Other diseases	Suicidal Ideation	Suicidal attempts	Marital status	Occupational status	Suicide modality	Time interval between last time heard/seen alive and body discovery	Time of death/ discovery	Days until autopsy	Body preservation state
**1**	Female	83	MDD	H	liver disease	no	no	widowed	unemployed	hanging	6 h	10:00 AM	4	abdomen discoloration
**2**	female	59	BD	H	no	yes	yes	single	unemployed	fall from height	witnessed	9:30 AM	2	fresh
**3**	female	64	MDD	H	no	yes	yes	single	unemployed	hanging	2 h	12:49 PM	2	fresh
**4**	female	40	BD	H	arthritis	yes	no	married	unemployed	fall from height	few minutes	8:10 AM	3	fresh
**5**	female	81	BD	H	cardiopathy	yes	no	widowed	unemployed	fall from height	witnessed	9:05 AM	3	fresh
**6**	male	54	BD	H	kidney disease	no	no	married	unemployed	fall from height	3 h	13:30 PM	4	abdomen discoloration
**7**	male	47	BD	H	high blood pressure	yes	no	married	unemployed	hanging	6 h	16:00 PM	4	abdomen discoloration
**8**	male	55	MDD	H	HIV	yes	yes	single	employed	sharp force trauma	8 h	20:20 PM	5	abdomen discoloration
**9**	female	40	BD	H	diverticulosis	yes	no	divorced	unemployed	fall from height	few minutes	20:25 PM	3	fresh
**10**	female	43	BD	H	inguinal hernia	no	no	single	unemployed	fall from height	witnessed	23:40 PM	4	fresh
**11**	female	43	MDD	H	no	no	no	married	employed	hanging	10 h	10:00 AM	4	abdomen discoloration
**12**	female	47	MDD	H	recurrent headches	no	no	married	employed	fall from height	8 h	7:30 AM	4	fresh
**13**	male	28	BD	H	asthma	yes	no	single	unemployed	fall from height	witnessed	17:20 PM	3	fresh
**14**	male	55	MDD	H	neck pain	no	no	divorced	unemployed	plastic bag	12 h	18:15 PM	2	abdomen discoloration
**15**	male	71	MDD	PP	benign prostatic hyperplasia	yes	yes	married	retired	hanging	7 h	8:00 AM	3	fresh
**16**	female	45	MDD	OPC	alcoholism and drug abuse	yes	yes	married	employed	hanging	4 h	7:40 AM	2	fresh
**17**	male	57	MDD	OPC	alcoholism and drug abuse	no	no	single	unemployed	gunshot wound	witnessed	23:37 PM	4	abdomen discoloration
**18**	female	59	MDD	PP	gastritis	yes	no	single	employed	chemical	10 h	16:20 PM	3	abdomen discoloration
**19**	male	62	MDD	OPC	arthrosis	no	no	married	unemployed	hanging	1 h	15:30 PM	4	fresh
**20**	male	43	MDD	OPC	chronic rhinitis	no	no	divorced	unemployed	fall from height	5 h	14:30 PM	4	abdomen discoloration
**21**	male	32	BD	PP	no	yes	no	single	unemployed	sharp force trauma	witnessed	16:50 PM	3	fresh
**22**	male	33	BD	OPC	no	yes	no	divorced	unemployed	fall from height	witnessed	13:30 PM	4	fresh

The average age of the men was 48.8 +/- 13 years, that of the women was 55 +/- 15 years. Twelve cases were people suffering from MDD (6 men and 6 women), the remaining cases were affected by BD (5 men and 5 women). The average age of all the people composing the MDD group was 57 +/- 14, while those of the people affected da BD was 45 years +/- 15 (p-value = 0.07). Breaking down by gender, the average age of the men with depression was 57 +/- 8 years, that of men with bipolar disorder 39 +/- 10 years (p-value = 0.01). The average age of women with depression was 57 +/- 14 years, that of women with bipolar disorder 52.5 +/- 16 years (p-value = 0.6).

Fourteen cases (63.5%), 6 people with MDD (4 women and 2 men) and 8 with BD (5 women and 3 men), were people treated as outpatients or inpatients in one of the two hospitals involved in this study; 5 cases (23%), 4 people with MDD (1 woman and 3 men) and 1 men with BD, were treated in outservices for mental health centers; 3 cases (13.5%), 2 people with MDD (1 woman and 1 men) and 1 men with BD, were treated by private psychiatrists. Overall, there were no statistically significant differences with regard to the type of pathology and the place of treatment (p-value = 0.4).

In all cases, the patients had been under treatment for at least one year. In 13 cases (59%) suicidal thoughts had already been expressed by 8 people with BD (4 men and 4 women) and by 5 people with MDD (2 men and 3 women). Regrading previous suicide attempts, there were 5 cases (about 23%), 1 woman with BD and 4 people with MDD (2 men and 2 women). In 5 cases, these were people who had already expressed suicidal thoughts and made anti-conservative gestures. Looking at the total number of suicidal thoughts and attempts in the two different disease groups, there was a statistically significant higher incidence in the BD group (p-value < 0.001). This statistical significance was also maintained when the subjects of the two groups were broken down by gender (p-value = 0.02).

None of the people selected had been diagnosed with cancer. Three people suffered from chronic pain conditions (recurrent headaches and joint pain). Two people were alcoholics and one person was also diagnosed with drug addiction. Besides that, none of the subjects selected in this study were diagnosed with any other psychiatric disorder based on the available medical documentation. With regard to comorbidities, there was no statistically significant difference between the MDD and BD groups (p-value = 0.8).

A total of 8 people (36.3%) were married (3 with BD and 5 with MDD), the remaining 14 (63.7%) were not married, including single, divorced or widowed people (7 with BD and 7 with MDD). Finally, 17 (72.3%) were unemployed (retired in one case) at the time of suicide (10 with BD and 7 with MDD), while 5 (22.7%) were employed (all with MDD). The details of this information are shown in Table 1. No statistically significant difference was found in these socio-demographic characteristics (p-value = 1 for the marital status and p-value = 0.07 for the occupational status).

Analysis of current psychotropic drug therapies, which in all cases had been unchanged for at least 7 days prior to death, revealed that all patients were prescribed with a daily-intake therapy, in the vast majority with a single administration per day and in 6 cases with multiple administrations throughout the day. Furthermore, more than one medication was prescribed in almost all cases (20 of 22 cases). Although it was not the aim of this study to assess the clinical appropriateness of psychiatric therapies, it should be noted that in some cases (i.e. at least in cases nos. 2, 14 and 17) the prescribed therapy may not have been appropriate for the pathology the patients were suffering from. However, this specific aspect is not further investigated or discussed.

**Fig. 2:**
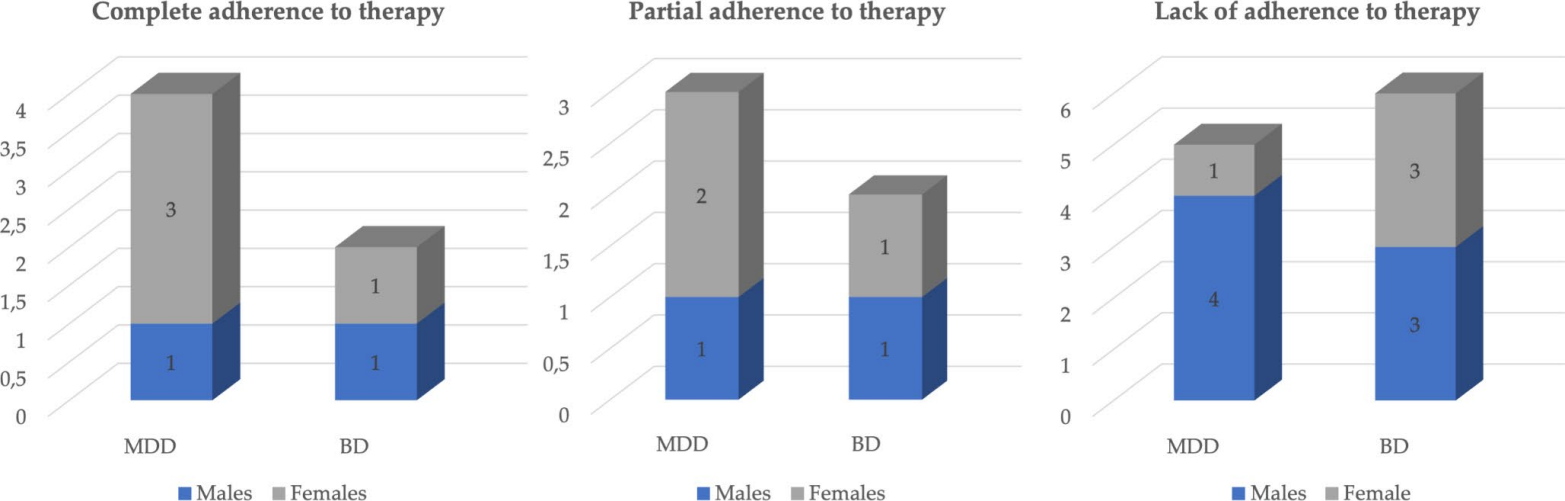
Detailed representation of the degree of adherence to psychiatric drug therapy by gender and pathology

Overall, the most frequently represented drug classes were: benzodiazepines, antidepressants, anxiolytics, antipsychotics, neuroleptics and mood stabilizers. The comparison of the toxicological analysis of the post-mortem samples with the therapy prescribed at the time of the suicide revealed the following: in only 6 cases (27.3%), 4 people with MDD and 2 with BD, did the drugs identified in the biological samples match qualitatively with those prescribed in the therapy (p-value = 0.4). In 5 cases (22.7%), 3 people with MDD and 2 with BD, there was only a partial match (p-value = 0.7), and finally in 11 cases (50%), 5 people with MDD and 6 with BD, no traces of the prescribed psychotropic drugs were found in the blood and/or urine sampled at the time of autopsy (p-value = 0.8). Stratifying the level of compliance by gender of the individuals in the two groups also did not yield statistically significant results. A detailed breakdown by gender and pathology of the subjects is shown in Fig. 2. For the sake of completeness, the half-lives of the drugs detected are also given.

The complete postmortem toxicological negativity concerned all main categories of psychotropic drugs, including lithium, for which concentrations in the physiological range were found. Further interesting results emerged from the quantitative evaluation of the toxicological findings. In 5 cases, not only were there fewer drugs in the blood than expected, but in 4 cases (2 with MDD and 2 with BD) concentrations below the therapeutic range were found (this was observed for nortriptyline, fluoxetine, clozapine and zolpidem). This was also observed in three cases (2 with MDD and 1 with BD) where there was instead total compliance between the prescribed medication and the toxicology findings (diazepam, lorazepam, quetiapine and temazepam).

The best compliance was observed in 4 women and 2 men who were referred to hospitals in 4 cases (2 with MDD and 2 with BD), to a out-patients mental health center in 1 case with MDD and to a private psychiatrist in 1 case with MDD. Of all these people, half were married and employed (3 cases, all with MDD), 2 were single and unemployed (all with BD) and 1 with MDD was retired and married.

On the other hand, partial treatment adherence was noted in 3 women and 2 men, 4 of whom were referred to hospitals (2 people with MDD and 2 with BD) and 1 case with MDD to a private psychiatrist. Most of these people were single and unemployed (3 cases 60%, 2 with MDD and 1 with BD).

In contrast, 7 men and 4 women were found to be totally non-compliant with treatment; of these 11 people, 6 were treated in the two hospitals (2 with MDD and 4 with BD), 4 in out-patients mental health centers (3 with MDD and 1 with BD) and 1 with BD by a private psychiatrist. Of all these individuals, most were single and unemployed (4 with BD and 3 with MDD, 63.5% in total); only 3 were married (1 with MDD and 2 with BD) and only 1 with MDD was employed.

For the sake of completeness, an attempt was made to correlate the various degrees of compliance (complete, partial, absent) with the gender of the patients (p-value = 0.45) and with the sociodemographic factors listed above, whereby no statistically significant differences were found (care facility: p-value = 0.58, marital status: p-value = 0.18, occupational status: p-value = 0.15).

The post-mortem toxicological analysis revealed further important aspects. For example, in 1 person (case no. 1), traces of a prescribed drug were detected only in the urine and not in the blood. In addition, 2 persons (case nos. 2 and 17) had ethyl alcohol in their blood, and 2 other persons (case nos. 10 and 16) had cocaine and its metabolites (case no. 10 in urine only). Finally, one person (case no. 18) had an unprescribed drug, citalopram, in their blood, which was taken in such large quantities for suicidal purposes that it was the cause of death. At the same time, in this individual, of the two remaining prescribed drugs, one was not taken at all and the other was below the therapeutic range. Details of the toxicological findings can be found in Table 2.

**Table 2:** Details of the post-mortem toxicological findings and of the half-lives of the detected substances

Patient	Diagnosis	Therpay	mg/die	Blood analysis	µg/mL	Urine analysis	µg/mL	Compliance	Blood therapeutic range	Half-life (h)
1 F, 83yo	MDD	Zolpidem	5	NEGATIVE		NEGATIVE		Partial		1.4–4.5^39^
		Nortriptyline	100	Nortriptyline	0.014	Nortriptyline	0.123		below range^33^	15–90^39^
		Amitriptyline	40	NEGATIVE		Amitriptyline	0.216			8–51^39^
		Lorazepam	3	Lorazepam	0.115	Lorazepam	0.482		in range^33–34^	9–16^39^
		Duloxetine	60	NEGATIVE		NEGATIVE				8–17^40^
2 F, 59yo	BD	Alprazolam	0.25	NEGATIVE		Missing sample		Partial		6–27^39^
		Fluoxetine	40	Fluoxetine	Traces				below range^33–34^	24–72^39^
		Mirtazapine	30	Mirtazapine	0.227				in range^35^	20–40^39^
		Triazolam	0.25	NEGATIVE						1.8–3.9^39^
		Flurazepam	30							1–3^39^
		Delorazepam	2							80–115^41^
				*Ethyl alcohol*	0.36 g/L					
3 F, 64yo	MDD	Paroxetine	30	NEGATIVE		Missing sample		Absent		7–37^39^
		Amisulpride	20 × 2							11–27^39^
		Lorazepam	3							9–16^39^
4 F, 40yo	BD	Carbolithium	300	NEGATIVE		NEGATIVE		Absent	physiological range (17,6 µg/L)^36–37^	17–58^39^
		Valproic acid	300							8–12^39^
		Chlorpromazine	100							7–119^39^
		Flurazepam	30							1–3^39^
5 F, 81yo	BD	Clomipramine	25 × 3	NEGATIVE		NEGATIVE		Absent		12–36^39^
		Valproic acid	300							8–12^39^
		Flurazepam	30							1–3^39^
		Hydroxyne	25							13–27^39^
6 M, 54yo	BD	Haloperidol		NEGATIVE		NEGATIVE		Absent		14–41^39^
		Valproic acid	500							8–12^39^
7 M, 47yo	BD	Clozapine	50	Clozapine	0.31	Clozapine	0.42	Partial	below range^33–34^	6–17^39^
		Paliperidone	depot	NEGATIVE		NEGATIVE				23 approx^42^
8 M, 55yo	MDD	Sertraline	50	NEGATIVE		NEGATIVE		Absent		22–36^39^
9 F, 40yo	BD	Carbolithium	300	NEGATIVE		NEGATIVE		Absent	physiological range (13,9 µg/L)^36–37^	17–58^39^
		Valproic acid	300							8–12^39^
		Haloperidol	5							14–41^39^
10 F, 43yo	BD	Bromazepam	3 × 2	Bromazepam	0.08	Bromazepam	0.1	Total	in range^33^	8–19^39^
		Clotiapine	25	Clotiapine	0.06	Clotiapine	0.14		in range^38^	4–7^43^
						*Benzoylecgonine*	Traces			
						*Cocaine*	Traces			
						*Ecgonine* *methyl ester*	Traces			
11 F, 43yo	MDD	Diazepam	4	Diazepam	0.04	Diazepam	0.05	Total	below range^33–34^	21–37^39^
		Sertraline	25	Sertraline	0.06	Sertraline	0.13		in range^33–34^	22–36^39^
		Promazine	120	Promazine	0.01	Promazine	0.03		in range^33–34^	7–17^39^
		Temazepam	12	Temazepam	0.02	Temazepam	0.09		in range^33–34^	3–13^39^
		Lorazepam	3	Lorazepam	Tracce	Lorazepam	Traces		below range^33–34^	9–16^39^
12 F, 47yo	MDD	Quetiapine	600	Quetiapine	0.07	Missing sample		Total	below range^33–34^	2.7–9.3^39^
13 M, 28yo	BD	Diazepam	5	Diazepam	0.07	Diazepam	0.04	Total	below range^33–34^	21–37^39^
		Temazepam	14	Temazepam	Traces	Temazepam	0.12		below range^33–34^	3–13^39^
14 M, 55yo	MDD	Bromazepam	3 × 2	NEGATIVE		NEGATIVE		Partial		8–19^39^
		Diazepam	4							21–37^39^
		Lorazepam	3			Lorazepam	0.19			9–16^39^
15 M, 71yo	MDD	Lorazepam	3	Lorazepam	0.192	Lorazepam	0.308	Total	in range^33–34^	9–16^39^
16 F, 45yo	MDD	Sertralina	50	Sertralina	0.13	Sertralina	0.05	Total	in range^33–34^	22–36^39^
				*Benzoilecgonine*	0.71	*Benzoilecgonina*	2.06			
				*Cocaine*	0.57	*Cocaina*	1.37			
17 M, 57yo	MDD	Diazepam	5	NEGATIVE		NEGATIVE		Absent		21–37^39^
		Lorazepam	3							9–16^39^
				*Ethyl alcohol*	4.18 g/L					
18 F, 59yo	MDD	Alprazolam	0.25	Alprazolam	0.032	Alprazolam	0.004	Partial	in range^33–34^	6–27^39^
		Zolpidem	10	Zolpidem	0.040	Zolpidem	1.622		below range^33–34^	1.4–4.5^39^
		Sertraline	25	NEGATIVE		NEGATIVE				22–36^39^
				*Citalopram*	4.502	*Citalopram*	38.301		overdose	
19 M, 62yo	MDD	Bromazepam	1 × 2	NEGATIVE		NEGATIVE		Absent		8–19^39^
		Paroxetine	20							7–37^39^
		Levosulpiride	10 gtt x 2							6–8^44^
		Lorazepam	2.5							9–16^39^
20 M, 43yo	MDD	Venlafaxine	75 × 3	NEGATIVE		NEGATIVE		Absent		3–7^39^
		Haloperidol	0,5 gtt							14–41^39^
		Lorazepam	3							9–16^39^
21 M, 32yo	BD	Fluoxetine	40	NEGATIVE		NEGATIVE		Absent		24–72^39^
		Flurazepam	30							1–3^39^
22 M, 33yo	BD	Valproic acid	300	NEGATIVE		NEGATIVE		Absent		8–12^39^
		Flurazepam	30							1–3^39^
		Fluoxetine	20							24–72^39^

## Discussions

Nowadays, it is a common experience, which is also repeatedly emphasised in the literature, that a large number of suicides occur in persons who are under medical supervision and are apparently taking psychiatric drug treatment [9, 45, 46]. This phenomenon has not yet been adequately addressed in the literature, which is why it was deemed necessary to conduct this pilot study aimed at analysing postmortem biological samples from individuals who died by suicide, suffered from a mood disorder and were prescribed pharmacological therapy in order to assess the victims’ adherence to therapy based on the blood levels of medications.

The first major hurdle was to select only those subjects who had been autopsied at the Institute of Forensic Medicine in Milan and whose last prescribed drug therapy could be determined. This posed a number of difficulties, as the relatives themselves often did not know whether the deceased was taking medication, what medication it was or in which care facility the deceased was treated for the psychiatric disorder. This should not be underestimated, as it may indicate a lack of awareness on the part of relatives [47] and/or a general underestimation of depression and its effects, as can also be found in the literature [48]. Furthermore, it is possible that psychiatric illnesses are still a taboo in today’s society [49]. However, we were able to select a total of 12 individuals with MDD (6 men and 6 women) and 10 with bipolar disorder (5 men and 5 women). Although the risk of suicide is higher in individuals with bipolar disorder, depression is more common in the general population [6, 8], which is also reflected in the case records of this study. The age of the victims ranged from 28 to 83 years, with an average age of 49 years for the men and 55 years for the women.

This is in line whith what is already known in the literature, namely that patients with psychiatric disorders who die by suicide are on average younger than the general population [50], for whom the peak in Italy is after the age of 60 [51]. Another finding that is consistent with the literature data is that the average age of people with bipolar disorder who died by suicide was lower than for people with depression. This is indeed a consequence of both a younger onset of illness and a higher risk of suicide with a higher number of suicide attempts in bipolar disorder [6, 52]. Indeed, statistically significant differences were found between the mean age of men with BD and those with MDD (p-value = 0.01), with the former being younger, and in the higher number of suicidal thoughts and attempts in individuals with BD (p-value = 0.01). Moreover, although it is known that the male gender is more frequently affected by suicide [53], in our case records men and women were equally affected. However, it must be point out that the case studies were selected based on the inclusion criteria and are therefore not representative of the totality of these events.

As far as the core of the study is concerned, the most important results related precisely to the adherence of the selected subjects to the prescribed drug therapy. In only 6 cases (27.3%) was there a qualitative match between the medications identified in the biological samples and the drugs prescribed in the therapy. In 5 cases (22.7%) there was only a partial match, and in the remaining 11 cases (50%) no traces of the prescribed psychotropic drugs or their metabolites were found in the blood and/or urine collected at autopsy. This applied to all drug classes, especially benzodiazepines, antidepressants, anxiolytics, antipsychotics and neuroleptics. And even when these were present, they were often below the therapeutic range [33–38]. This affected both depression and bipolar disorder to a very similar extent, with the latter slightly outweighing the former. However, there were no statistically significant differences in terms of the type of pathology and the degree of adherence to treatment, which was a predictable result due to the limited sample size.

Overall, the best compliance was observed in 4 women and 2 men, 4 of whom referred to hospitals, 1 to an out-patients mental health center and 1 to a private psychiatrist. Half of these people were married and employed. In contrast, a predominance of single and/or unemployed subjects was observed among those with no or partial compliance with therapy. However, given the limed sample size, no statistically significant differences were found, even though some p-valus, particularly in relation to marital status (p-value = 0.18) and occupational status (p-value = 0.15), appeared to be closer to the significance thresold.

These specific aspects are not particularly surprising, as they are consistent with the literature on patients with psychiatric disorders [29, 52] and are also known with regard to compliance with other and non-psychiatric drug therapies [54]. In fact, the literature reports that single people (including divorced and widowed) tend not to adhere to the prescribed therapy, probably because there is no caregiver to ensure that the medication is taken regularly [55–57]. Incidentally, this trend seems to be particularly pronounced among men. At the same time, a correlation between unemployment and a lack of adherence to treatment has also been established, whether due to a lack of money or a low level of education [58, 59]. In addition, marital status (single people) and occupational status (unemployment) are themselves recognized as risk factors for suicide [60–62]. So these are aspects that should always be examined when investigating people who have died by suicide.

Obviously, suicide is a complex phenomenon that can vary considerably between genders, age groups, geographical areas and socio-political conditions [9], but as mentioned earlier, abstinence and/or non-adherence to drug treatment is thought to exacerbate depressive symptoms and may lead to an increase in suicidal ideation [63, 64]. However, although adherence to drug therapy is certainly a protective factor that reduces the risk of suicide, it is not able to eliminate it completely [65]. In any case, it is clear that this is a very critical scenario, as more than 70% of people suffering from a psychiatric disorder and being treated with a prescribed drug therapy only partially adhere to it or do not adhere to it at all. It is well known in the literature that patients with psychiatric disorders, especially schizophrenia, have low compliance, which is why various strategies such as the use of extended-release drugs and increased monitoring are used [66]. However, this study shows that similar compliance problems also exist in individuals with mood disorders, which must inevitably lead to clinical and care considerations aimed at better understanding the phenomenon and implementing new strategies to improve compliance in these patients.

In this study, the investigation of selected cases was furtherly extended by the contextual analysis of blood and urine samples in order to obtain an even more complete overview. The half-life of drugs is indeed one of the aspects that should always be considered when it comes to adherence to drug therapy based on the assessment of their blood levels. In this study, numerous drugs with different half-lives ranging from a few hours to several days were detected [39–44]. However, considering that all subjects selected in this study were supposed to be on daily drug therapy, it can be concluded that drugs with an extended half-life have most likely not been taken for some time. This is all the more true for the drugs for which double or triple daily administration was intended, such as amisulpride, clomipramine, bromazepine, venlafaxine and levosulpiride. The urine anlysis confirmed this, as it was often completely negative [30]. In addition, in two cases (no. 1 and no. 14) traces of drugs with a long half-life (amitriptyline and lorazepam, respectively) were only detected in the urine and not in the blood. This is to be interpreted as an expression of irregular and not even recent intake by the patient [33, 34]. In contrast, for drugs with a shorter half-life, where the time of ingestion by the subjects who later died by suicide is not known (although it is usually in the morning or evening), urine tests were crucial and would certainly have shown the presence of recently ingested drugs that are no longer present in the blood [30]. In this sense, since no urine was available in case no. 3, the hypothesis of partial adherence to treatment cannot be ruled out a priori, even if this is very unlikely, since the medications in question have a relatively long half-life (especially amisulpride), traces of which should have been found in the blood if the intake had been recent.

In all cases where the blood levels of medicines were below the therapeutic range, this was most likely due to improper intaking or incorrect dosing in the patients concerned. Indeed, another consideration is that many medications are subject to genetic variation, which can lead to a more or less rapid metabolism of the substances [67]. For clinical purposes, genotyping analyses on living individuals are possible in order to adapt drug treatment accordingly [68], but these have not yet been applied in a post-mortem forensic context. In order to better assess treatment compliance, other factors were taken into account in this study, such as the exact dosage prescribed, the concentration of the medications in the blood, their half-life and their presence or absence in the urine of well-preserved bodies.

In addition, ethyl alcohol was detected in the blood of two people and cocaine and its metabolites in two other cases. These findings are quite relevant, as the consumption of drugs of abuse is known to play a dual role: addiction increases the risk of suicide in the long term, while acute intoxication dramatically increases the risk of suicide by removing inhibitions and reducing coping strategies to resolve suicidal thoughts [69, 70]. In addition, the molecules of the ingested illicit substances can interact and influence the correct pharmacokinetics and pharmacodynamics of the ongoing drug therapy [71]. Finally, one person had an unprescribed medication in their blood that was taken for suicidal purposes, and furthermore, of the three prescribed medications, one was not taken at all and the other appeared to be below the therapeutic range. In this case, therefore, the two aspects of non-compliance with the therapy and the toxic effect of medication taken in inappropriate quantities come together. This illustrates the pathological-forensic and clinical complexity that can occur in such cases. Therefore, a toxicologic examination is essential in all psychiatric patients in general and even more so in suicide cases, as it provides crucial information on whether the cause of death is due to intoxication, side effects of medical treatment or possibly lack of compliance [72, 73].

More generally, the issue of people with psychiatric disorders deserves further attention. They are known to have an increased risk of premature death and a high mortality rate from non-natural causes, which increases with concomitant illicit drug use [74, 75]. Indeed, given the circumstances typically associated with deaths in this population, decedents with psychiatric disorders are often referred for forensic autopsy [76]. At this point, it is therefore appropriate to consider the forensic autopsy as a valuable tool for the comprehensive investigation of the death of suicidal persons with psychiatric disorders. In this context, the progressive decrease in the number of autopsies in many countries is a worrying trend that must be halted as soon as possible [77], as autopsy is a pivotal tool for the protection of public health in its broadest sense.

### Strengths and limitations

The present study has some limitations as it is very challenging to take into account all factors that might be related to suicide. For example, we do not know the degree of inner suffering of the people selected in this study, the degree of insight into the disease, any non-pharmacological therapies (e.g. cognitive behavioral therapies) and other social and economic factors. Precisely for this reason, the study was approached as objectively as possible, focusing on postmortem toxicological analysis and comparison with the drug therapy prescribed and ongoing at the time of suicide. The objectivity of this approach is a strength of the study, which does not aim to prove or measure the relationship between treatment adherence and suicidality or to investigate the clinical correctness of the prescribed therapy, but rather to point out the critical issues that currently exist and perhaps still receive too little attention. The sample selected is modest, but reflects the difficulty of meeting the inclusion criteria, which nevertheless made it possible to select only cases that were suitable for the study and had verifiable clinical information. These strcit exclusion criteria justify the small number of cases selected compared to the large number of people who died by suicide and had a mood disorder undergoing autopsy. It should also be considered that the two hospitals we have collaborated with are among the leading hospitals in Milan, but there are many others in the city. It is therefore quite conceivable that many of the people who could potentially have been included, but whose health data we were unable to find, were treated in other hospitals. An extension of the study to other hospitals would certainly be a future research perspective and could also overcome the limitation resulting from the fact that the small number of cases in this study weakens the significance of the statistical analyses. The fact that the p-values are close to the significance threshold in some cases suggests that larger samples could be useful to confirm the observed trends. Finally, the hair samples of the subjects selected in this study were not analyzed as they were no longer available retrospectively. This did not allow us to examine chronic and past exposure to drugs and other substances, although this was not the aim of the study; future prospective research in this field should take this possibility into account. On the other hand, urine analysis allowed the toxicologic window of exploration to be expanded, providing a check compared with blood analysus alone. In general, some of the limitations mentioned above are due to the fact that this was a pilot study on a topic not yet addressed in the literature, for whose approach and mode of investigation there was no reference.

## Conclusions

Our data should contribute to the current state of knowledge in order to gain insights for optimizing the clinical management of patients with mood disorders and suicidality. Actually, a very bleak scenario emerged: the vast majority of people with depression or bipolar disorder who died by suicide were not taking their medication as prescribed. Irrespective of the extent to which there is a connection between non-compliance and suicide, it is a fact that the therapy was not adequately taken by patients. Considering that good compliance is considered a protective factor for suicide risk, it is clear that something went wrong in the cases examined in this study, which represent only a small percentage of those who are affected by a psychiatric disorder and die by suicide. In all probability, then, this is the so-called tip of the iceberg of a much larger and widespread phenomenon in today’s society. A decisive call to action is needed here, as these are deaths that could perhaps have been avoided. Much has already been done in recent years, but further efforts seem necessary. For example, clinicians and caregivers must be increasingly sensitized not to underestimate a depressive symptom and not to assume that treatment is taken timely and as prescribed. With this in mind, suicide prevention education programs in primary care should always include a specific plan to reinforce and assess treatment adherence in subjects at risk of suicidal behaviors, not only through clinical discussions and discouragement of substance use, but also by encouraging regular surveillance. Although monitoring adherence to medication treatment in outpatients is still a major challenge in psychiatric clinical practice, this could be another step forward. In the meantime, the study of postmortem medication adherence in suicidal individuals should be considered a useful technique for evaluating the effectiveness of specific medical interventions and highlighting existing critical problems. This pilot study could thus form the basis for the implementation of an epidemiologic monitoring and clinical feedback protocol for pharmacologically treated psychiatric patients who have died by suicide for identifying new approaches to suicide prevention. Such prospective surveillance would build a bridge for direct discussions and synergetic cooperation between clinicians and forensic pathologists. And perhaps bringing together these two worlds, which normally speak little or not at all to each other, could be a successful strategy. On the other hand, it should never be forgotten that much can be learned from the dead to contribute to the well-being of the living, especially if they are affected by psychiatric disorders and thus belong to one of the most vulnerable population groups.

## Electronic supplementary material

Below is the link to the electronic supplementary material.

Supplementary Material 1

## Data Availability

All the data have been reported in the manuscript.
