# Supplementary material for: Mood disorders and suicide: pilot study on postmortem toxicologic evidence and adherence to psychiatric therapy by determining blood levels of medications
Source: Int J Legal Med. 2024 Sep 14;139(1):319–34. doi: 10.1007/s00414-024-03327-8 (PMC11732864; doi:10.1007/s00414-024-03327-8)
Supplement: Supplementary file 1 — Supplementary Material 1 [file 414_2024_3327_MOESM1_ESM.docx]

**Supplementary Material S1**

The following chemical-toxicological tests were performed on the blood and urine samples according to the methods routinely used at the Institute of Forensic Medicine of the University of Milan:

- analysis for volatile organic compounds including alcoholometric determination.

- analysis for non-volatile organic substances with the aim of detecting narcotic and/or psychotropic substances, as well as a general address for the search for other substances of toxicological interest.

- Quantitative determination of the substances detected.

***Analysis for volatile compounds***

The presence of volatile organic compounds can be detected directly on biological and non-biological material by gas chromatographic analysis of a gas sample taken from an unconnected container (vial) into which the sample to be analysed has been placed.

This technique, known as “headspace",” is based on Henry's law, which states that in a closed system at constant temperature, the partial pressure of a component in the gas phase is proportional to its concentration in the underlying phase. The components of the injected mixture are separated according to the different affinity of the components to the stationary phase of the gas chromatography column or to the carrier gas.

The analyses to evaluate the profile of the volatile components were carried out under the following conditions:

- Thermofisher GC Trace 1300 Gas Chromatograph;
- Column: STABILWAX-MS 15m x 0.25mmID x 0.25µm f.t. (Restek);
- Oven temperature program: 35°C 5 minutes, from 35°C to 60°C with an increment of 4°C/min, from 60°C to 160°C with an increment of 6°C/min, from 160°C to 200°C with an increment of 20°C/min, finally holds at 200°C for 20 minutes;
- Injector temperature: 250°C constant temperature;
- Split: 1:10;
- Source and transfer line temperature: 250 and 210° C respectively;
- Carrier gas: helium 1 ml/min flow.

The internal standard method was used for the quantitative determination of volatile compounds (terbutanol 0.5 g/L in water).

To determine the blood alcohol level, a calibration curve was prepared with standard solutions of ethanol in water (Cerilliant®) in concentrations of 0.25 - 0.5 - 1 - 2 - 3 g/L, each mixed with the same internal standard.

The quantitative determination of ethanol was carried out according to the following formula:

Ethyl alcohol (g/L) = C/R x 0.5, where:

C = ratio of the area of the analyte in the sample to that of the internal standard.

R = ratio of the peak area of the reference standard to that of the internal standard.

0.5 = concentration of ethyl alcohol in the reference standard, expressed in grammes/liter.

***Analysis for non-volatile compounds***

*Full-scan search with GC-MS*

The extracts obtained after solid phase extraction with 130 mg Bond Elut® Certify columns (Varian) were analyzed according to the following procedure. 0.5 mL of each sample was made up to a volume of 1 mL with double-distilled water, SKF525-A was added at a concentration of 1 mcg as an internal standard and 4 mL of phosphate buffer pH 6.88 was added. After vortex shaking and centrifugation for 10' at 3500 rpm, the resulting solutions were loaded onto the column, which had previously been adjusted with 2 mL methanol and 2 mL phosphate buffer pH 6.88.

After washing with 2 mL pH 6.88 buffer, 1.5 mL 0.01 M hydrochloric acid and 0.3 mL methanol, the columns were dried for 30 minutes. The samples were then eluted first with 2 mL of a mixture of chloroform and acetone in a 1:1 ratio, from which the acidic/neutral extract was obtained; a second elution with 1 mL of ethyl acetate in 2 % ammonia, followed by 1 mL of a mixture of dichloromethane and isopropanol in a ratio of 8:2 in 2 % ammonia, gave the basic extract.

The resulting extracts, which were dried in a rotary vacuum evaporator, were reconstituted with 100 mcL of methanol; 2 mcL of the resulting solution was analyzed by GC/MS.

An Agilent 6890 Network gas chromatograph with an Agilent 5977c mass detector and a Varian Chrompack CP-Sil8 CB lowbleed/MS capillary column with a length of 15 m, an inner diameter of 0.25 mm and an inner coating thickness of 0.25 mcm was used.

The analyses were carried out under the following operating conditions:

| Carrier: | helium at a pressure of 7 psi |
| --- | --- |
| Oven temperature: | 70°C isotherm for 2'; 70°C to 160°C with an increment of 40°C/min; 160°C to 290°C with an increment of 8°C/min and final isotherm for 2 minutes. |
| Injector temperature: | 270°C |
| Temperature detector: | 300°C |
| Scanning range: | 40-550 m/z |

***HPLC/MS-MS research acid-base extract***

The analytical protocol allows the detection of a large group of substances with acidic or basic behavior which, if present in the biological material, are separated by extractive procedures with suitable organic solvents.

The tests were carried out according to the methods described below in their essential parts.

*Sample preparation*

The extracts obtained after solid phase extraction with 130 mg Bond Elut® Certify columns (Varian) were analyzed according to the following procedure.

Lidocaine at a concentration of 1 mcg as an internal standard and 4 mL phosphate buffer pH 6 were added to the samples made up to a volume of 2 mL with distilled water. After vortex shaking and centrifugation for 10' at 3500 rpm, the resulting solutions were loaded onto the columns, which had previously been acclimatized with 2 mL methanol and 2 mL phosphate buffer pH 6.

After washing with 2 mL pH 6 buffer, 1.5 mL 0.01 M hydrochloric acid and 0.3 mL methanol, the columns were allowed to dry for 30 minutes. The samples were then eluted first with 2 mL of a mixture of chloroform and acetone in a 1:1 ratio, from which the acidic/neutral extract was obtained, and with a second elution with 1 mL of ethyl acetate in 2 % ammonia, followed by 1 mL of a mixture of dichloromethane and isopropanol in a ratio of 8:2 in 2 % ammonia, from which the basic extract was obtained.

The extracts thus obtained were dried in a rotary vacuum evaporator and reconstituted with 100 mcL of methanol; 2 mcL of the resulting solution was analyzed.

For this purpose, a FORTIS system was used under the following operating conditions: HPLC system (Thermo Fisher Scientific, San Jose, CA, USA) consisting of a quaternary Surveyor MS pump with a degasser, an automatic Surveyor AS sampler, a column oven, and a Rheodyne valve with a 20-μL loop.

A Synergi Hydro-RP reversed-phase HPLC column (150 × 2.0 mm, particle size 4 μm) with C18 precolumn (4 × 3.0 mm) (Phenomenex, Torrance, CA, USA) was used for chromatographic separation. Solvents A (formic acid in 0.1 M water) and B (MeOH) were the mobile phases used for the gradient. A Thermo Fortis (Thermo Scientific, San Jose, CA, USA) with a heated electrospray ionization source (HESI) was used as the detector. The capillary temperature and evaporator temperature were set to 350°C and 300°C, respectively, while the electrospray voltage was set to 3.50 kV in positive mode.

The full-scan acquisition was combined with an independent data acquisition mode (DIA) that provided MS2 spectra for confirmatory responses based on an inclusion list. The resolving power of the FS was set to 70,000 FWHM. A scan range of m/z 50-650 was selected based on our composite list. The quadrupole filtered the precursor ions with an isolation window of 2 m/z. The fragmentation of the precursor ions was optimized as ramp collision energy (10-60 eV).

***Analysis for lithium assessment***

*Chemicals and reagents*

All chemicals and reagents used in the preparation and analysis of the samples were of analytical grade.

Plastic containers were soaked in 10% v/v HNO3 for 24 hours and carefully rinsed with distilled water before the collection of the sample. Laboratory glassware used was as well soaked in 10% (v/v) HNO_3_ for 24 hours, rinsed with distilled water, dried, and checked for contamination prior to use.

*Sample preparation*

The sample for ICP-MS analysis was prepared by placing 0.5 mL of whole blood in 15 mL propylene tubes equipped with a screw cap (VWR laboratories) and digesting the sample in accordance with EPA DG-CL03 standard procedures using nitric acid (HNO_3_, 69% Hiperpur Solution) and hydrogen peroxide (H_2_O_2_, 30% Hiperpur Solution) supplied by Panreac Quimica SLU (Castellar del Valles, Barcelona, Spain). We used a Milli-Q system (Millipore, Merck KGaA, Darmstadt, Germany) to obtain purified water.

Calibration points were obtained by digesting blank matrices spiked with appropriate concentrations of lithium standard solution (Agilent Technologies) in aqueous solutions of 5% HNO_3_.

All the samples were then mineralized following the previously cited EPA digestion method.

We used an inductively coupled plasma mass spectrometer (Agilent 7500ce, Agilent Technologies, Santa Clara, CA, USA) equipped with a Cetac ASX-510 auto-sampler (Thermo Fisher Scientific, San Jose, CA, USA). Gases used in analysis (argon and helium) were 99.999% pure.

System optimization was assessed using a tuning solution consisting of Cs (cesium, 55), Co (cobalt, 27), Li (lithium, 3), Mg (magnesium, 12), Tl (thallium, 81), and Y (yttrium, 39) (Agilent Technologies, Palo Alto, CA, USA).

Internal standard solution used of the analysis was obtained from a stock Agilent Yttrium standard solution appropriately diluted with 1% (v/v) nitric acid until reaching a concentration of 1 µg/mL. The so obtained internal standard was added using a peristaltic pump.
